# Isotherm and Electrochemical Properties of Atrazine Sensing Using PVC/MIP: Effect of Porogenic Solvent Concentration Ratio

**DOI:** 10.3390/membranes11090657

**Published:** 2021-08-26

**Authors:** Nuur Fahanis Che Lah, Abdul Latif Ahmad, Siew Chun Low, Nur Dina Zaulkiflee

**Affiliations:** School of Chemical Engineering, Engineering Campus, Universiti Sains Malaysia, Nibong Tebal 14300, Malaysia; nuurfahanis@usm.my (N.F.C.L.); chsclow@usm.my (S.C.L.); nurdinazaulkiflee@gmail.com (N.D.Z.)

**Keywords:** molecular imprinting, porogen, isotherm study, electrochemical sensor

## Abstract

Widespread atrazine use is associated with an increasing incidence of contamination of drinking water. Thus, a biosensor using molecularly imprinted polymers (MIPs) was developed to detect the amount of atrazine in water to ensure prevention of exposure levels that could lead to reproductive effects in living organisms. In this study, the influence of the porogen on the selectivity of MIPs was investigated. The porogen plays a pivotal role in molecular imprinting as it affects the physical properties and governs the prepolymerization complex of the resulting polymer, which in turn firmly defines the recognition properties of the resulting molecularly imprinted polymer (MIP). Therefore, bulk MIPs against atrazine (Atr) were synthesized based on methacrylic acid (MAA) as a functional monomer and ethyleneglycol dimethacrylate (EGDMA) as a crosslinker; they were prepared in toluene and dimethyl sulfoxide (DMSO). The imprinting factor, binding capacity, and structural stability were evaluated using the respective porogenic solvents. Along with the characterization of the morphology of the obtained polymers via SEM and BET analysis, the kinetic and adsorption analyses were demonstrated and verified. The highest imprinting factor, binding capacity, and the highest structural stability were found to be on polymer synthesized in a medium of MAA and EGDMA, which contained 90% toluene and 10% DMSO as porogen. Moreover, the response for Atr concentrations by the PVC-based electrochemical sensor was found to be at a detection limit of 0.0049 μM (S/N = 3). The sensor proved to be an effective sensor with high sensitivity and low Limit of Detection (LOD) for Atr detection. The construction of the sensor will act as a baseline for a fully functionalized membrane sensor.

## 1. Introduction

Atrazine has recently been one of the most frequently detected drinking water-contaminating pesticides. Atrazine is a commonly used herbicide to prevent pre-emergence broadleaf and grassy weeds where it is mainly used in agriculture, with the largest use being seen in maize, sorghum, and sugar cane. It is an endocrine disruptor, an agent that can alter the natural hormonal system of living organisms. Therefore, detecting the amount of atrazine in water is crucial to avoid exposure levels that could cause reproductive damage in living organisms. Thin-layer chromatography [1], micellar electrokinetic chromatography [2], multivariate electronic spectroscopy [3], voltametric competitive immunosensor [4], high-performance liquid chromatography [5], and gas chromatography–mass spectrometry [6] are some of the technologies used to detect atrazine. These methods, however, have substantial drawbacks due to their high cost, complexity, and large material consumption due to the wide range of chemicals and equipment required for the process.

Thus, a pseudo functional material for specific recognition of target compounds by imprinting the functional groups and conformation of template molecules, known as Molecular Imprinted Polymer (MIP), has received outstanding research and application in various fields such as separation process [7], pharmaceuticals [8], sensors [9,10,11], and drug delivery [12,13,14,15,16]. This is due to its characteristics; MIPs are robust, stable, relatively inexpensive, resistant to a wide range of pH, humidity, and temperature and can be synthesized for analytes for which no natural antibody exists. In order to fully develop a stable and functionalized membrane electrochemical sensor, the fundamentals and mechanisms of imprinted polymer need to be fully optimized. 

The mechanism strategy relies on the MIP cavities that retain the chemical structure allowing the template to lock into the cavity [17]. In the existence of the molecule template of the target analyte, the crosslinking and functional monomers are copolymerized during the polymerization/crosslinking process. In choosing the monomers, the potential to interact with the template molecule’s functional groups is prioritized. As a result, the cavities preserve their “memory” for the target analyte after the extraction of the template molecule from the polymer. In most cases for the imprinted polymer, a porogenic solvent or nonreactive polymer is added to enhance overall MIP porosity, which helps to facilitate the template diffusion through an MIP.

The porogen mixture depends on the polarity and solubility parameter of a monomer, polymer, and porogen and the interaction between them. The excellent performances of MIP depend on the use of various organic solvents as porogen [18] such as toluene, odecyl alcohol/cyclohexanol, chloroform, and acetone to tune the porosity and morphology. Generally, there are three types of solvent quality, which are good, poor, and theta solvent. The solvent was divided based on the interaction between the solvent with the monomer. The use of mixtures of two types of solvent (good and poor solvents) enabled the generation of the flow through pores [19]. The preference for polymerization to occur inside monomer-swollen nuclei increased as the relative amount of a poor solvent for building polymer chains increased [20]. Furthermore, the production of larger globules that agglomerate is envisaged, resulting in increased pore diameters. While on the contrary, the polar porogen systems’ typical destabilising effect on monomer–template interactions can be addressed by utilising an aprotic less polar porogen. 

As proved in a report [21] elsewhere, small alterations in the concentrations or porogen type of the polymerization mixture may have an effect on the pore size distribution leading to extreme flow resistance. In spite of the fact that MIP binding and selectivity in batch rebinding mode may not be greatly influenced by macroporosity, mass transfer kinetics associated with polymer porosity may be important in applications such as controlled release and template delivery. In our studies to date, we have demonstrated that the MIP imprinting factor for target atrazine increases in the order of acetone < chloroform < DMSO [22] as a coporogen together with toluene. In order to maximize the potential for additional future applications, the pore formation of the polymer was altered by manipulation of the ratio between solvents to attain an efficient, permeable, and stable system. In comparison to a single porogen system, adding DMSO as a cosolvent allows for the production of polymers with large pores. The mixture of porogens proved to be a dominant factor in influencing the polymers’ ability to release and rebind the template thus defining the phase behaviour of the resultant polymer. According to Young’s rule, the relative solvent composition for mixtures variety may lead to the solvent tenability [23]. In order to maximize the potential for additional future applications, the ratio between these solvents is also a crucial step and should be optimized to attain efficiency, permeability, and stability. Although this step is critical, the efforts put forth in this research area are very limited [19]. This work is part of our efforts to investigate the feasibility of functional MIP systems as part of the PVC sensor construction. In extending the use of DMSO as a coporogen with toluene from previous studies, we therefore anticipated a trend of imprinting capability by manipulating the volume of porogen ratio between the two solvents. These findings can give useful hints for designing the synthesis of novel MIP in a high monomer concentration. Aiming to follow the fourth industrial revolution strategy, an electrochemical set up was incorporated into the detection mechanism together with the imprinted polymer. 

## 2. Materials and Methods

### 2.1. Chemicals and Materials

Methacrylic acid (MAA), ethylene glycol dimethacrylate (EGDMA), Atrazine (Atr), and Dimethyl sulfoxide (DMSO) were purchased from Sigma-Aldrich (St. Louis, MO, USA). 2,2-azobisisobutyronitrile (AIBN) was purchased from Molekula (Darlington, UK). Toluene was purchased from Fisher (Leicestershire, UK). All chemicals were commercially available as analytical reagents.

### 2.2. Preparation of Atr-MIP

The atrazine MIP was prepared in a 7 mL glass tube by dissolving the atrazine (Atr) template in the porogen (with varying ratios of DMSO and toluene as shown in Table 1) [10,22]. The mixture was then added to a functional monomer Methacrylic acid (MAA), crosslinker Ethylene Glycol Dimethacrylate (EGDMA), and initiator (Azobisisobutyronitrile (AIBN)). The mol ratio of each component between monomer:crosslinker:template was maintained at 40:15:1 throughout the experiment. Nitrogen gas was purged for 5 min and sealed to optimise the polymerization reaction. The tube was left in a water bath at 60 °C for 20 h for the polymerization to occur.

**Figure 1:**
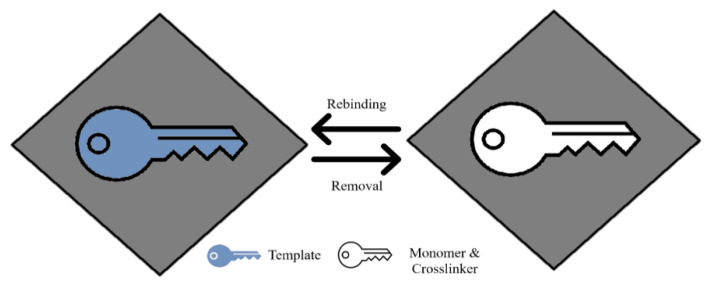
Concept of molecularly imprinted polymers and the adsorption capacity of nonimprinted polymers.

Concurrently, a nonimprinted polymer (NIP) was prepared in a uniform manner except without the addition of the template as a control. Then, the polymer was smashed, ground, and dried in a desiccator for 24 h. Following polymerization, the template was extracted from the polymers using methanol [20], and the test was carried out by immersing the template molecule in the Atr binding solution. It was then left for drying at room temperature for 24 h. As shown in Figure 1, this extraction-binding approach simplified the concept of molecular imprinting.

Two types of solvent, i.e., methanol and water, were investigated as a washing and binding solvent. NIP was also tested in the same procedure as a control. The recovery of the template atrazine in the washing fraction was evaluated.

### 2.3. Guest Binding and Morphology of Atr-Imprinted Polymer

The rebinding analysis of MIP and NIP for Atr were studied by adding 5 mL of Atr solution at an initial concentration of 20 ppm to 25 mg of polymer. The vials were agitated in a basic variable-speed digital orbital shaker (IKA TS130 Basic, Rawang Integrated Industrial Park, Rawang, Selangor, Malaysia) at room temperature for 120 min. The sample was drawn and separated by using a 4 mm syringe filter to ensure no polymer was transferred to the sampling bottle, which may have clogged the HPLC. The volume of Atr adsorbed by MIP and NIP was verified by injecting 10 µL of each analyte to a C18 analytical column of high-performance liquid chromatography (HPLC) (Agilent Technologies, Santa Clara, CA, USA), which was controlled at 40 °C. Atr was quantified at a wavelength of 230 nm with an effluent of methanol and water ratio of 55:45 at a flow rate of 0.4 mL·min^−1^. Then the polymers were characterized. All experiments were reported with the standard deviation of the average value after three times of sample reading. The adsorption capacity, *Q* (mg g^−1^ of polymer) of Atr bound to the polymer was determined by the following equation:

(1) $$Q=\frac{\left(C_{0}-C_{t}\right)V}{W}$$

where *C*_0_ and *C_t_* (mg L^−1^) are the initial and residual Atr concentrations at time *t*, respectively. *V*(L) is the volume of Atr solution and *W* (g) is the mass of MIP and NIP (Renkecz et al. 2014, Ang et al. 2016). The imprinting factor of the polymer was calculated by the following equation:

(2) $$IF=\frac{Q_{MIP}}{Q_{NIP}}$$

where $Q_{MIP}$ is adsorption capacity of molecular imprinted polymers while $Q_{NIP}$ is the adsorption capacity of non-imprinted polymers.

The pore characterization of the polymer was conducted by using a nitrogen adsorption–desorption measurement at 77 K (Micromeritics ASAP 2020, Norcross, GA, USA). The Brunauer–Emmett–Teller (BET) theory was utilized to compare the specific surface areas of the polymer. The Barrett–Joyner–Halenda (BJH) analysis was utilized to verify the pore diameters of MIP. On the other hand, the mesopore volumes were developed through the association of a t-plot by Harkins and Jura. The surface micrographs analysis of both MIP and NIP were conducted by tabletop scanning electron microscope (SEM) (Hitachi TM3000, Tokyo, Japan), with resolution in the micrometer (µm) range at a voltage of 5 kV.

### 2.4. Electrochemical Application of MIPs as Sensor

**Figure 2:**
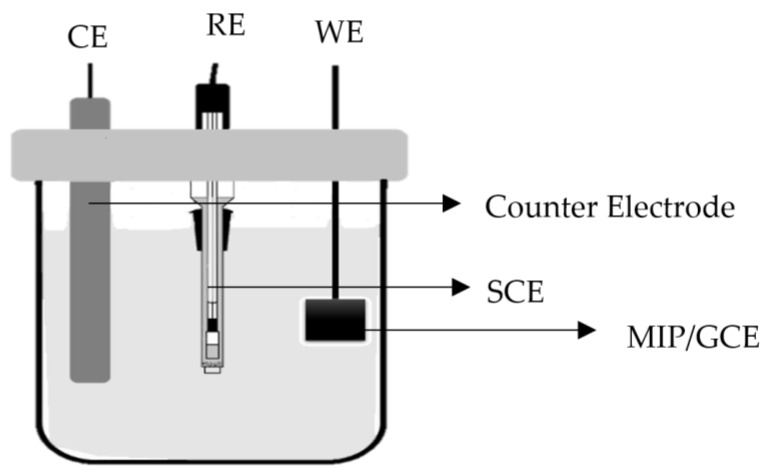
Electrochemical cell setup with three types of electrodes (i) Counter Electrode (platinum wire), (ii) Reference Electrode (SCE), and (iii) Working Electrode (MIP/GCE).

The working electrode (PVC/MIP/GCE electrode), the counter electrode (platinum wire), and the reference electrode (SCE) were utilised in a conventional three-electrode cell assembly. The cyclic voltammograms were documented under the closed-circuit condition and the voltage was changed from −2.0 V to 1.5 V against SCE in forward and reverse scans at the scan rate of 40 mV·s^−1^. The kinetics of the heterogeneous electron transfer were determined by conducting the experiments at different scanning rates of 20, 40, 60, and 100 mV·s^−1^ using a 1 mmol·L^−1^ K_4_[Fe (CN)_6_] solution as the supporting electrolyte solution. The sensor construction and setup were prepared according to our previous work [22] at room temperature (25 °C), as illustrated in Figure 2. To prepare the sensor electrode for subsequent usage, 0.5 cm^2^ of graphite felt was soaked in 5 mL of ethanol. Amidst the electrode and the polymer, 0.5ml of 4% *w*/*w* PVC in NMP was utilised as a binder. To polymerize the PVC and attach it to the electrode, 1 mL of acetone was used as the solution. The electrode was then dried for 24 h at room temperature. All binding was done with a fixed amount of 25 mg polymer (after the template was removed).

### 2.5. Theoretical Isotherm Modelling

Four adsorption isotherms were studied which were Linear, Langmuir, Freundlich and Jovanovic.

(3) $$\mathrm{Linear}\;\mathrm{adsorption}\;\mathrm{isotherm}\;:\;B_{e}=a_{L}+b_{L}\cdot F_{e}$$

(4) $$\mathrm{Langmuir}\;\mathrm{adsorption}\;\mathrm{isotherm}\;:\;B_{e}=\frac{N\cdot K\cdot F_{e}}{1+K\cdot F_{e}}$$

(5) $$\mathrm{Freundlich}:\;B_{e}=k_{F}\cdot F_{e}^{\frac{1}{n_{F}}}$$

(6) $$\mathrm{Jovanovic}\;\mathrm{adsorption}\;\mathrm{isotherm}:\;B_{e}=N_{J}\left(1-e^{-k_{J}\cdot F_{e}}\right)$$

For the kinetic isotherm, The Lagergen first order model is expressed as:

(7) $$B_{t}=B_{e}\left(1-e^{-k_{1}\cdot t}\right)$$

where *k*_1_ (g g^−1^ h^−1^) is the rate constant of the first order model and *t* is the time.

The pseudo second order model is expressed as:

(8) $$B_{t}=\frac{B_{e}^{2}\cdot k_{2}\cdot t}{B_{e}\cdot k_{2}\cdot t+1}$$

where *B_e_* and *B_t_* refer to adsorption amount (mg mg^−1^) of the polymer at the equilibrium and time *t* (min). *k_2_* is the constant of the kinetic model.

The Elovic kinetic model is expressed as:

(9) $$B_{t}=\frac{\ln\left(\alpha \cdot \beta \right)}{\beta }+\frac{1}{\beta }\ln t$$

where *B_t_* is the amount of Atrazine adsorbed at time *t* (min) and *α* (g/g h) is the initial adsorption rate and *β* (g/g) is the desorption constant during experiment. These seven models were selected to be fitted to the experimental data. The best model fitted was chosen based on the statistic evaluation that is described below. 

In minimizing the error that may appear when utilizing only one statistical method [24], three pertinent statistical methods were linked together in a more thorough and effective statistic tool which is the sum of normalized errors (SNE) [25]. 

Another two classical statistical evaluation methods used were the Student’s *t*-test and the *F*-test. In the *t*-test:

(10) $$t=\frac{Z}{s}=\frac{\left(\bar{X}-\mu \right)}{\left(\frac{\sigma }{\sqrt{n}}\right)}$$

where $\bar{X}$ is the sample mean from a sample of size *n* comprising *X*_1_, *X*_2_, …, *X_n_*, *σ* is the standard deviation, *μ* is the population mean, and *s* is the standard error of the mean. The *t*-test is used to examine if the sets of data differ substantially from one another [23], while in a one-way analysis of variance, the *F*-test is used to determine if the predicted values of a quantitative variable within various predefined groups differ from one another [24]. The highest value of the test resulted in a better fit of the model to the experimental data. 

## 3. Results

### 3.1. Selection of Washing and Binding Solvent

**Figure 3:**
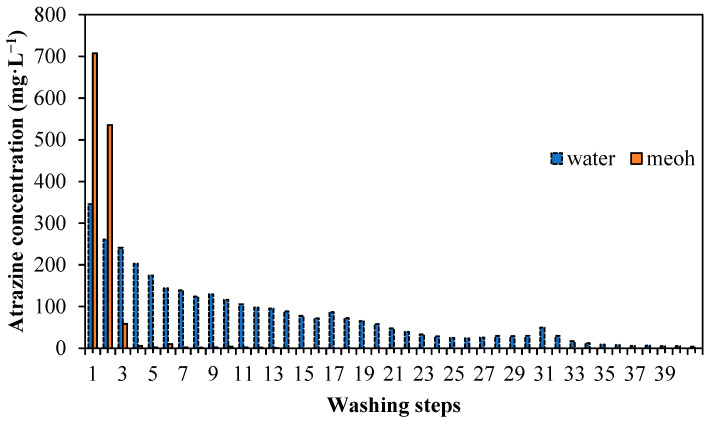
Washing steps in different solvents.

As the atrazine was strongly adsorbed on the MIP, it should be released in the washing step to leave the cavities for rebinding steps to take place. As shown in Figure 3, the analyte was easily washed out by using methanol as a washing solvent. Only four washing steps were needed to wash out the atrazine from the imprinted polymer when methanol was used. However, when water was used as the washing solvent, the steps increased to almost 40 to clear the atrazine from the polymer system. For MIP, all the interferences were also more easily removed by using methanol rather than water. Methanol would be a favourable solvent for the extraction of the template from the polymer due to the lower number of washing steps required to easily break the attachment between the template and the binding sites. Methanol gave a higher recovery in the study up to 99.8% for imprinted polymer. 

Table 2 summarizes the findings for both solvents used as the binding solution. Several researchers have suggested that some morphological changes occur in MIP during molecular recognition of the target analyte [26,27]. Thus, the degree of the imprinting factor could have been affected depending on the solvent used as the binding solution. When water was used as the testing medium, the binding capacity of MIP was found to be greater than when methanol was used. This is because atrazine is less soluble in water (34.7 mg·L^−1^) compared to in methanol (22.7 × 10^3^ mg·L^−1^). This led to the higher attraction of atrazine from the testing solution to MIP/NIP, which was caused by the weaker interaction between water and atrazine. Thus, atrazine was more easily transported to the imprinted site, resulting in greater binding. Atrazine’s higher solubility disrupted the interaction of the imprinted sites with the analyte. By using water as the binding solution, the polymer lost in the process was almost 1.4 times higher compared to when methanol was used as the binding solvent. However, the binding capacity was almost 22 times higher when water was used as the aqueous medium to dissolve Atr (target analyte). Therefore, water was selected as the percentage of mass loss was significantly acceptable. The aqueous medium used to dissolve Atr throughout this study was the water solution.

### 3.2. Imprinting and Binding Capacity

**Figure 4:**
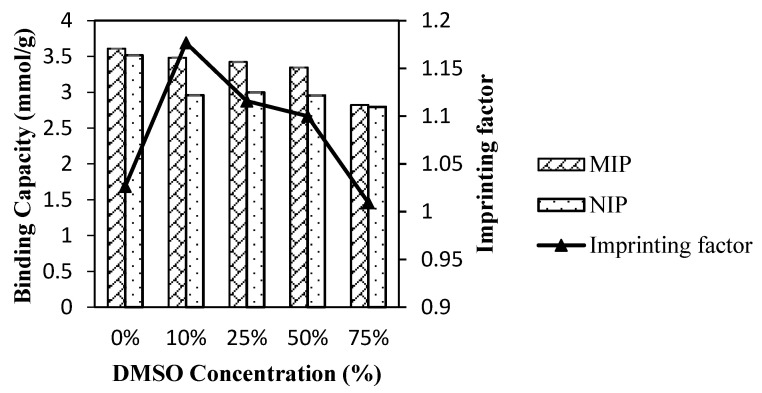
Adsorbed amounts of Atr by MIP and NIP for different porogenic solvent concentrations.

Selective binding was most pronounced in the case of the system with the use of DMSO as a coporogen producing lower nonspecific binding to the nonimprinted material thus increasing the imprinting factor for the polymer. Thus, the effect of coporogen concentration in the system was studied to investigate the effect of the solvent concentration ratio on the binding. Figure 4 shows the adsorbed amounts of atrazine by MIP and NIP for different porogenic solvent concentrations.

As depicted in Figure 4, the binding capacity decreased with the increment of DMSO concentration for both imprinted and nonimprinted polymer. The same trend can be observed for the imprinting factor and the binding capacity. This demonstrated that the addition of DMSO caused an increment of template configuration within the resultant polymer as well as reduce the binding ability. However, as the concentration of DMSO was increased, the cavities reduced with much lower binding capacity. The imprinted polymer showed a larger gap (2% to 15% of decrement) of binding capacity as the concentration increased compared to nonimprinted polymer (0.2% to 5% of decrement). It proved that DMSO affected the monomer–template interaction in the polymer construction. Bird and Herdes proved that by adding DMSO in a binary mixture of porogen in TNT–MAA molecular imprinted polymer system, it increased the interaction of the MAA molecule with the template as the cluster size decreased [20]. This is crucial to calculate the quality of the template–monomer complex and eventually predict the template–monomer rebinding capabilities.

### 3.3. Morphology of MIPs

**Figure 5:**
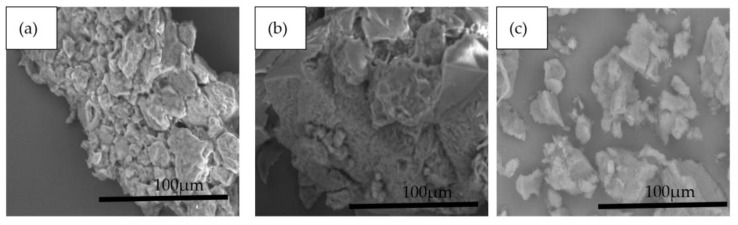
SEM images of imprinted polymer with (**a**) 10% DMSO, (**b**) 25% DMSO, and (**c**) 75% DMSO (Mag: 1.0K).

Figure 5 demonstrates the SEM images of the polymers with various fraction of DMSO. As the concentration increased, the polymer monolith became denser with a smaller pore diameter, as shown in Table 3. The shape of the monolith changed drastically with no more globules observed when the porogen is 100% of the toluene used [22]. Other factors besides the solubility parameters that affected the porous properties of the polymer may have affected the morphology of the polymer. The evaporation rates for toluene and DMSO are about 1.9 and 0.026, respectively (evaporation rate of n-butyl acetate is standardized as 1.0). Moreover, the vapor pressures for toluene and DMSO are about 21.75 and 0.46 mm Hg at 20 °C. Therefore, toluene evaporates much faster than other solvent even at room temperature. However, this phenomenon might be minimal as the system was conducted in a closed system. In addition, the phase separation during polymerization occurs earlier with toluene, and this results in the formation of larger pores.

### 3.4. Surface Area Measurements MIPs

Table 3 tabulates the surface area, pore volume, and pore diameter of the polymer obtained whereas Table 1 shows the solubility parameter for the porogen mixture. As mentioned earlier, the resulting morphology of MIP depended on the difference in solubility parameters between the monomer and the porogen. However, the effect of solubility parameter seemed to contribute less to the pore construction as no specific trend can be observed for changes in surface area, pore volume, and the average pore diameter when the concentration of DMSO was altered. It was proven that the solubility parameters were not the main contributors to the porosity of the polymer monolith when these two porogen were used in the system. 

In this study, toluene proved to be a good pore forming agent in MAA/EGDMA based polymers with a high imprinting factor, which is in line with findings from Yi, et. al. [28]. This supports our finding on the minimum usage of DMSO and maximum usage of toluene as the solvent affects the template–monomer interaction in improving the selectivity of the polymer. Higher levels of good cosolvent contribute to the colloidal stability of the primary particles formed. Additionally, at lower solvency the homocoagulation period of primary particles is prolonged, resulting in final irregular shapes and surfaces [22].

### 3.5. Sorption Isotherm 

**Figure 6:**
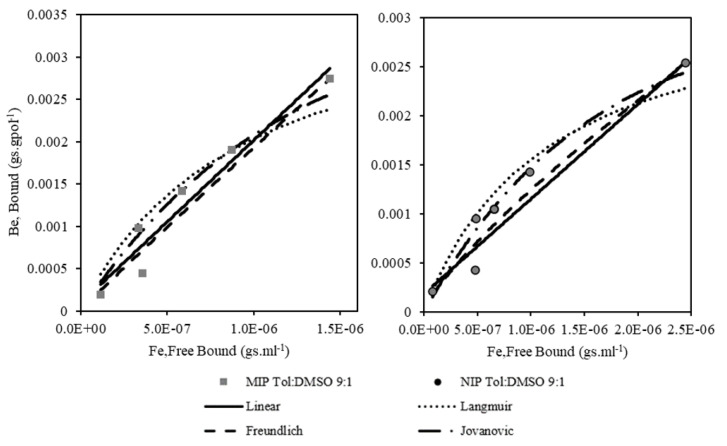
Fitting the experimental data at equilibrium with Linear, Langmuir, Freundlich, and Jovanovic adsorption models.

Four adsorption models for equilibrium experiment and three adsorption models for kinetic experiments are discussed in this section. Figure 6 presents the equilibrium data, with the amount of Atr bound on MIP (B_e_) versus the free Atr that remained in the solution (F_e_). It was then fitted with Linear, Langmuir, Freundlich, and Jovanovic models. Figure 6 is based on the fitting of adsorption isotherm for MIP and NIP prepared using 90% of toluene and 10% of DMSO as the porogenic solvent. 

The parameters of the isotherms model obtained from the model fitting were listed in Table 4. For the Langmuir and Jovanovic isotherms, it was observed that the quality of imprinting, expressed as IF, can be correlated with the energy (rate) of adsorption (K) and corresponding binding site density (N_J_) values which simultaneously gave the highest value from the MIP Tol:DMSO 9:1 sample. 

The statistical evaluation of the adsorption isotherms is presented in Table 5 to assist in choosing the best-fitted isotherm to the system. The bold numbers represent the maximum values for each statistical method ($1-{\bar{R}}^{2}$, $r\chi^{2}$ and SEE) that were used in the calculation of the normalization step in obtaining the Sum of Normalized Errors (SNE) values. The minimum SNE values showed the type of polymer the adsorption model fits the best. 

The adsorption models that fit best with the experimental data are illustrated by looking at the SNE column of Table 5, which consists of the sum of MIP errors and sum of NIP errors. The minimum value of the sum of SNE corresponded to the Jovanovic model for both MIPs and NIPs (bold). The order of all four models based on the minimum SNE criteria for both MIPs and NIPs was as follows: Jovanovic > Linear > Freundlich > Langmuir.

N_J_ is the corresponding binding site density and K_J_ is the adsorption constant, as interpreted by the Jovanovic adsorption isotherm model. Two aspects of the Langmuir hypothesis are considered in the Jovanovic adsorption model, which are identical adsorption sites and monolayer adsorption. Meanwhile in the case of Jovanovic isotherm, MIP Tol: DMSO 9:1 system had the lowest K_J_ and the highest N_J_ and IF value from the MIP series. This reinforced the notion that a high binding capacity (N) or a high adsorption constant (K) alone were insufficient to characterise an MIP with good adsorption properties when individually taken. The trend of the binding capacity and the adsorption constant for the Jovanovic adsorption isotherm completely contradicted the findings for the Langmuir adsorption isotherm. As the binding site density and the adsorption constant values of both models were compared, the relation of N > N_J_ and K < K_J_ for MIP and NIP was observed. Under the assumption of existence between these two isotherm models, the interaction of the free and bound molecules would lead to a decrease in the binding sites’ density and an increase in the adsorption constant. The system also occurred on a heterogeneous binding site rather than homogeneous as the Freundlich hypothesis seemed to be more favorable than the Langmuir isotherm. By understanding this binding characteristic of the polymer, further modification for improving the system can be made.

In addition, two other statistical tests, namely the *F*-test and the Student’s *t*-test, were examined to predict the best-fitting isotherm model. From Table 6, both of the classic statistical tests also show that the Jovanovic isotherm portrayed the best isotherm for polymer adsorption. 

In summary, the successful interpretation of the dynamic adsorptive separation of solute from solution onto a sorbent depends upon a good depiction of the equilibrium separation between the two phases. By graphically plotting solid-phase concentration against residual liquid-phase concentration, it depicted that the Jovanovic equilibrium adsorption isotherm gave the best description for the imprinted polymer with 90% toluene and 10% DMSO as the porogen. 

### 3.6. Kinetic Modelling

**Figure 7:**
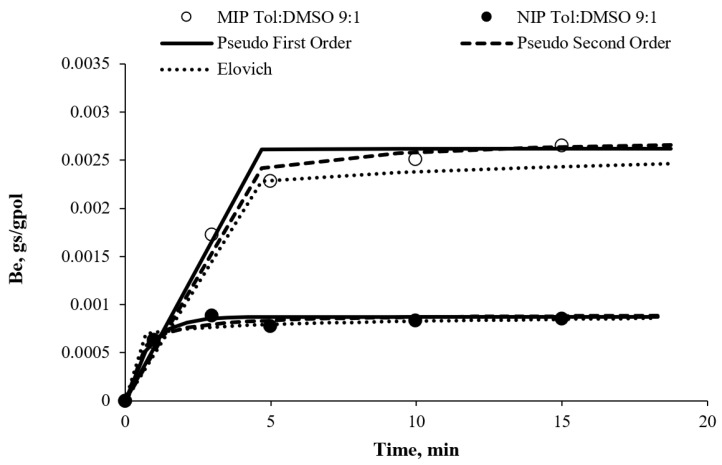
Kinetic evolution of MIP Tol:DMSO 9:1 and NIP Tol:DMSO 9:1 in 20 ppm of Atr concentration.

In order to evaluate the adsorption kinetics of atrazine on MIPs and NIPs, Lagergen first order (a first-order rate equation to describe the kinetic process of liquid–solid adsorption), pseudo second order, and Elovich kinetic models (to prove that the sorption was based on the chemisorption process) were chosen to fit the experimental data. All the data presented in Figure 7 show the kinetic evolution of MIP and NIP Tol:DMSO 9:1 adsorbent. Table 7 presents the kinetic parameters that were obtained for all the kinetic models presented in all experimental settings. The first 0.3 h data are presented in Figure 7 to show the most significant trend and to prove that a fast and reliable sensor for detection was successfully constructed. The fitting curves were shown only for MIPs and NIPs that was tested with 20 ppm of Atr for a more readable figure. 

An observation from Table 6 shows that the MIP adsorbent had higher *k*_1_ values than NIP. A similar trend can be observed for the constant rate of pseudo second order that was expressed as *k_2_·B_e_*. The α parameter of Elovich model, which signified the adsorption rate, was higher for MIP in comparison with NIP while the β parameter, which signified the desorption rate, was smaller for MIP than NIP. Generally, all three kinetics models underlined that the MIP had higher adsorption properties than the NIP. The error for the kinetic data was calculated by the sum of individual errors for each sorbent at all four initial concentrations in a similar manner to the adsorption isotherm error analysis. It suggested that the rate of the solute uptake changed with time was proportional to the difference in saturation concentration (q_e_) and the amount of Atr adsorbed (q_t_) with time. Compared to NIP, the highest rate constant of the first model, *k*_1_ values proved that the MIP Tol: DMSO 9:1 was a better adsorbent. 

### 3.7. Electrochemical Properties of MIPs as a Sensor

**Figure 8:**
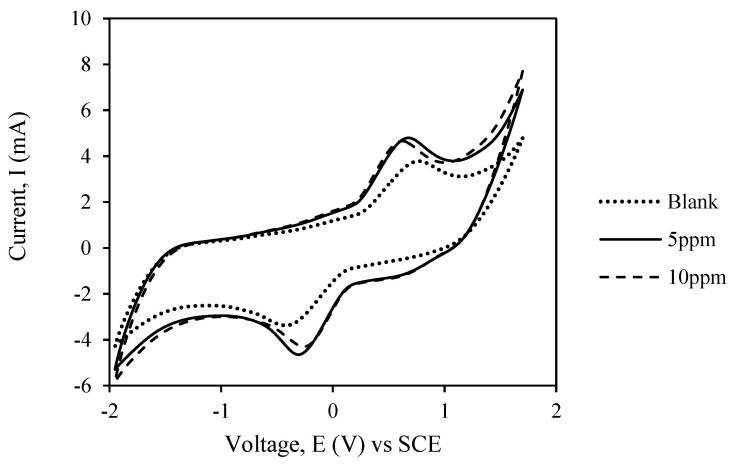
Cyclic voltammograms of the bare electrode in potassium ferrocyanide solution containing an increasing concentration of atrazine. The scan rate was 40 mV s^−1^.

The imprinted polymer was assembled with a graphite felt electrode (MIP/GFE) which acted as an electrochemical transducer, to develop an atrazine-based electrochemical sensor. Due to its high conductivity, PVC was chosen as the binder. Figure 8 displays the cathodic currents measured at the bare graphite felt electrode in the absence of Atr and for an increasing concentration of atrazine at scan rate of 40 mV·s^−1^. The calculated current clearly increased with the addition of atrazine to the system. However, when the concentration was increased from 5 ppm to 10 ppm not much difference in the peaks could be observed. This result was in agreement with those obtained in the case of atrazine reduction on platinum electrode reported elsewhere [29]. Both oxidation and reduction peaks were embedded in a large wave and could be observed clearly. This observation implied that atrazine reduction, which was likely to be a two-electron process, was an irreversible process. An irreversible process involves a slow electron transfer rate, which needed an extreme electrode potential to drive the electron transfer and register current on the potentiostat [30]. 

**Figure 9:**
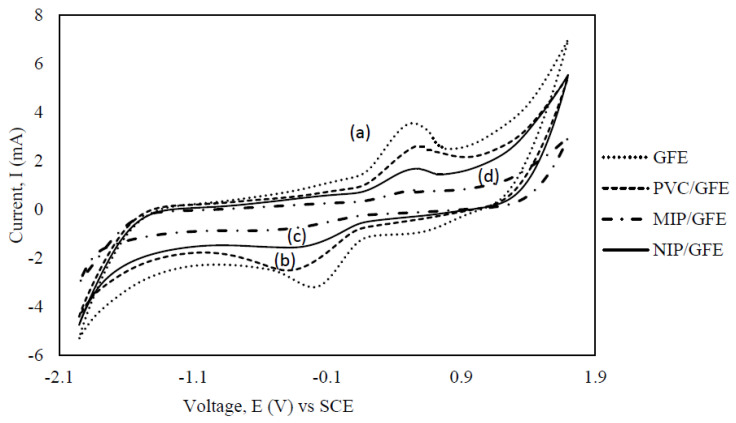
Cyclic voltammograms obtained using the (**a**) GFE (**b**) PVC/GFE (**c**) NIP/GFE, and (**d**) a MIP/GFE in 20 ppm of atrazine.

Figure 9 shows the cyclic voltammograms of the bare GFE, PVC/GFE, MIP/GFE, and NIP/GFE in potassium ferrocyanide solution. It can be seen that electrochemical reaction occurred in the potential range of −0.5 V to 0.5 V for scan rates of 40 mVs^−1^ resulting in different redox peaks, respectively. Compared to the bare GFE, which had a pair of well-shaped redox peaks (curve a), weaker peaks appeared as the PVC was bound to the electrode as shown by curve b in Figure 9. A clear decrement in the peak current density was observed on the graphite felt electrode after it was bound with PVC. This can be ascribed to the change of the surface structure of the electrode, reducing the porosity and resulting in slower diffusion to the electrode surface. An obvious decrement of the current density could be observed when the nonimprinted and imprinted polymer, bonded to the electrode’s surface with PVC, were tested. This finding proved that the polymer was successfully integrated with the electrode by PVC as the binder. 

A decrement of current density in NIP may also be due to the unspecific adsorption of atrazine to NIP. The presence of pendent MAA residues along the polymer chains or at the polymer matrix surface may be able to establish hydrogen bonds with atrazine. Once the NIP/GFE was introduced to 20 ppm of atrazine, just a pair of weaker peaks (comparing to curve a and b) appeared (curve c). Even in the presence of atrazine, the NIP redox probe was able to detect some electron transfer that occurred in the system. It showed that the binding of the atrazine to the NIP was not enough to block the active pore on the electrode. As expected, only weak redox peaks were observed with the MIP/GFE (curve d) over the potential range, which indicated that a compact of scarcely conductive MIP polymer was coated on the electrode and blocked the contact of the active pore with the electrode surface. It revealed that some channels and cavities were recombined with atrazine, giving limited chances for the electron transfer of the redox probe on the MIP/GFE.

A similar finding was observed by Pardieu, et al. [29] in 10^−3^ mol·L^−1^ atrazine detection at −0.5 V versus the reference electrode at the scan rate of 25 mVs^−1^. A substantial decrease in current was detected when atrazine was added to the solution. It was associated with the conformational variations taking place in the polymer throughout its redox activity, which involved two forms of polymer chains: the polyaromatic and the polyquinonic chains. However, in their case of NIP system, no alteration of the cyclic voltammogram was seen upon addition of atrazine. Whilst, in our study, some modification was still be able to be detected on the NIP system as we were using a different construction method of the electrode. This is due to the fact that our NIP system was still able to capture the nonspecific binding of the atrazine. Some improvement needed to overcome this circumstance. 

Yan, et al. [31] compared the cyclic voltammograms of oxidation reduction probe solution (ORPS) on the bare glassy carbon electrode (GCE), NIP/GCE, and MIP/GCE. While a pair of well-shaped redox peaks was observed with the bare GCE, no redox peaks were displayed when the MIP/GCE electrode was used before the Atr was removed. After the templates were extracted from the MIP/GCE, the pair of redox peaks could be observed once again. These redox peaks occurred due to the removal of the template, leaving the channels free for the electron transfer of the redox probe on the MIP/GCE. In this case, they introduced the target analyte separately before testing it out while this study was run in situ together with the existence of the target analyte in the electrolyte. In this study, the recombination of polymer and the Atr limited the chances of electron transfer of the redox probe on the MIP/GCE. Although the method used slightly varied, similar findings were observed between this study and the one by Yan, et al. [31] in that the redox peaks were observed after the target analyte was re-introduced to the imprinted polymer. Positive testing of MIP sensor towards the atrazine proved that the set up can be used while maintaining the specific binding of the imprinting polymer characteristic.

#### 3.7.1. Cyclic Voltammetry Studies

**Figure 10:**
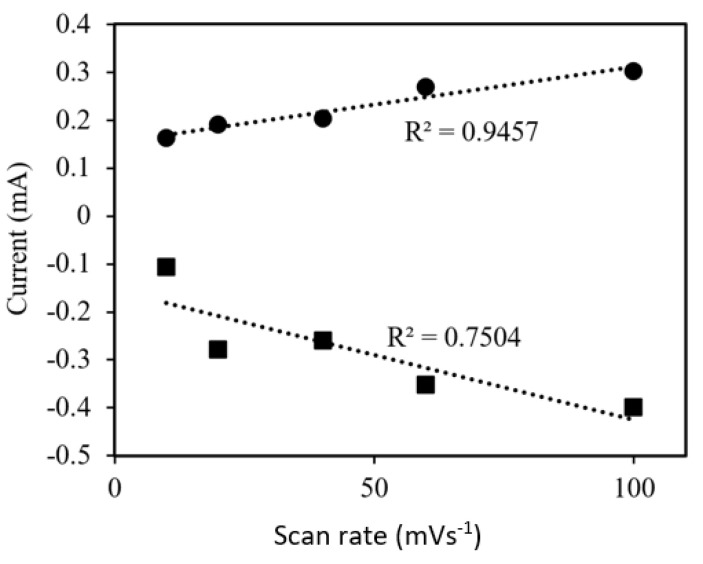
Relationship between scan rate and anodic ● (Ipa) and cathodic ■ (Ipc) peak currents.

The effect of scan rate on the MIP/GFE and NIP/GFE was investigated. The study was conducted in a 20 ppm atrazine concentration. From Figure 10, it can be seen that both the cathodic current (*I_pc_*) and anodic current (*I_pa_*) were proportional to the scan rate and can be expressed as: *I_pc_* (mA) = −0.0027υ−0.1549 (R^2^ = 0.7504) and *I_pa_* (mA) = 0.0016υ + 0.1533 (R^2^ = 0.9457) (here, υ is the scan rate in the unit of mV/s), suggesting a typical surface controlled electrochemical behaviour. Similar findings were observed by Yan, et al. [31] as an electrochemical sensor for isocarbophos detection. This phenomenon occurred due to the migration of molecules from the bulk aqueous phase to the interface and were characterized by the rate of molecule transport from the bulk to the interface. 

The characterization of the system can be demonstrated by the analysis of the redox peaks with different scanning rates. The ratio of the oxidation peak electric current to its corresponding reduction counterpart, I_pa_/I_pc_ was about 0.679 to 1.529. The peak ratio decreased with the increase in scanning rate. Moreover, the peak potential separation, Δ𝐸𝑃 = 𝐸_𝑝𝑎_−𝐸_𝑝𝑐__,_ was between 60 and 177 mV. It increased with the variation of scanning rate. The results obtained in Figure 8 are evidence that the redox process followed a quasi-reversible reaction rather than reversible reaction or irreversible reaction. The possibility of quasi-reversible and irreversible system indicated that the mechanism of the current was controlled by both the charge transfer and mass transport that involved the redox reaction in the electrolyte solution and the mass transport of atrazine to the imprinted sites.

**Figure 11:**
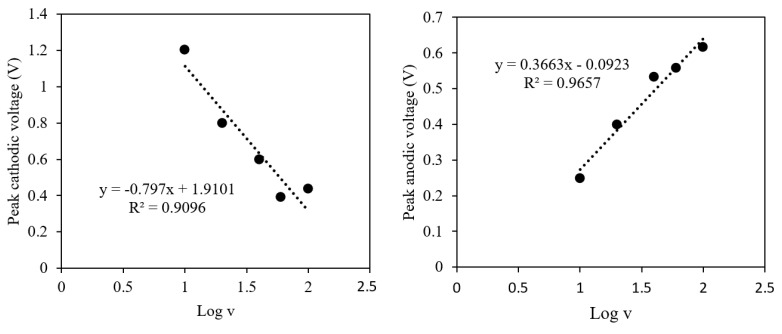
Plot of (**left**) cathodic and (**right**) anodic voltage versus log of scan rate for the imprinted polymer.

The peak characterization obtained from the CVs can be used to determine the heterogeneous electron transfer rate constant, 𝑘*_d_*. The 𝑘*_d_* value was determined from an equation proposed by Nicholson and Shain [32]. The peak of anodic and cathodic voltage against Log of scan rate of CV as were plotted as represented in Figure 11. A linear relationship of this plot gave a slope, m, and intercept for value of K. The heterogeneous electron transfer rate constant, 𝑘*_d_*, was solved by using the parameters’ values shown in Table 8.

Accordingly, the value of the heterogeneous electron transfer, 𝑘*_d,_* for the anodic process was 0.0661 cm·s^−1^ and the cathodic process, 0.0195 cm·s^−1^. In comparison to the standard rate of heterogeneous electron transfer rate in Table 7, these values were between the ranges of 0.020 > 𝑘*_d_* > 5.0 × 10^−5^ cm·s^−1^ which were eligible to be considered as a quasi-reversible system. 

#### 3.7.2. Limit of Detection

Experiments were carried out to check the electrochemical behavior of atrazine on the modified electrode in different initial concentrations of atrazine. When MIP/GFE was used for a working electrode, the more catalytic ability and the less peak potential difference were obtained against the redox probe. The current of the redox peaks (I_p_) increased with increasing atrazine concentration, suggesting that more and more atrazine was recognized by the imprinted sites in the MIP/GFE. It can be associated with the conformational variations occurring in the imprinted polymer during its redox activity. The imprinted polymer chain can twist to adapt to the steric strain, interaction between the carboxyl group in the print molecule and the amino group in the atrazine, inflicted by the existence of atrazine linked to the pendent acetic acid groups of the monomer [29]. It also demonstrated that the MIP/GFE can give rise to recognition sites complimentary to the molecular shape, size, and functionality of atrazine molecules. 

The linear regression equation was calculated from the redox peaks as I (mA) = 0.286 − 0.1879 C (M) with a correlation coefficient of 0.9974. The limit of detection (LOD) was estimated to be at 0.0049 μM (S/N = 3). Visibly, the suggested electrode showed potential for application in the observing of atrazine with its comparatively low LOD and fast response. A difference in the performance of various electrochemical sensors for the determination of atrazine is reviewed in Table 9.

## 4. Conclusions

The synergistic effect of the usage of both good and bad solvents produced a resultant polymer that could capture the advantages of the solvents in the particle formation. The highest imprinting factor, binding capacity, and the highest structural stability have been found to be on a polymer synthesized in a medium of MAA and EGDMA, which contained 90% toluene and 10% DMSO as a porogen. In this case, toluene improved the intermolecular crosslinking of the particle formation whereas DMSO functioned as the porogen to form an extremely crosslinked cluster of particle globules. A large number of imprinted sites were found on the vigorously heterogeneous binding centres, which then created a monolayer with an identical adsorption site. The kinetic sorption properties of the polymer were obtained and the rate of the solute uptake changes with time showed a proportional relationship to the difference in saturation concentration (qe) of the solute uptake and the amount of Atr adsorbed (qt) with time. The equilibrium and kinetic sorption data acquired will help in development of an effective Atr sensing technique on the molecularly imprinted polymer. The response for Atr concentrations by the electrochemical sensor was found to be at a detection limit of 4.99 nM (S/N = 3). The fundamental understanding of the PVC/MIP sensor construction through porogen ratio manipulation opened up a few possibilities that may improve the sensor efficacy.

## Figures and Tables

**Table 1:** Hildebrand solubility parameter of the porogen.

Material	Hildebrand Solubility Parameter (J^1/2^/cm^3/2^)
100% Toluene	18.30
90% Toluene + 10% DMSO	19.11
75% Toluene + 25% DMSO	20.33
50% Toluene + 50% DMSO	22.45
25% Toluene + 75% DMSO	24.38

**Table 2:** Absorbed amounts of Atr by MIP and NIP and imprinting factors for different binding solutions. Note: Polymer synthesized using a constant molar ratio of template:crosslinker:monomer.

Binding Solution	Sample	Binding Capacity (mg.g^−1^ of Polymer)	Imprinting Factor	Mass Loss (%)	Atrazine Solubility (mg·L^−1^)
Methanol	MIP	0.2899	0.9463	15.8	22.7 × 10^3^
	NIP	0.3063		8.6	
Water	MIP	6.5790	1.3744	22.3	34.7
	NIP	4.7866		16.3	

**Table 3:** BET specific surface area, specific pore volume, and average pore diameter for different porogenic solvent concentrations.

Sample with DifFerent Porogen (% DMSO)	Specific Surface Area (m^2^ g^−1^)	Specific Pore Volume (cm^3^ g^−1^)	Average Pore Diameter (nm)
10	237.5415	0.02885	0.5666
25	141.1416	0.01218	0.5535
50	126.5631	0.05837	0.5327
75	218.1825	0.03480	0.5512

**Table 4:** The parameters of applied adsorption isotherms for MIP and NIP in 10% DMSO of coporogen.

Model	Par.	MIP Tol:DMSO 9:1	NIP Tol:DMSO 9:1
Linear	a_L_	3.08 × 10^−5^	1.85 × 10^−4^
	b_L_	1.89 × 10^3^	9.67 × 10^2^
Langmuir	N	1.05 × 10^6^	8.57 × 10^5^
	K	3.97 × 10^−3^	3.37 × 10^−3^
Freundlich	k_F_	1.13 × 10^4^	7.42 × 10^1^
	n_F_	1.04 × 10^0^	1.25 × 10^0^
Jovanovic	N_J_	3.60 × 10^−3^	3.05 × 10^−3^
	K_J_	8.59 × 10^5^	6.57 × 10^5^

**Table 5:** The errors of the adsorption isotherms.

Model	Error	MIP Tol:DMSO 9:1	NIP Tol:DMSO 9:1
Linear	$1-{\bar{R}}^{2}$	0.0643	0.0622
	$r\chi^{2}$	8.49 × 10^−8^	6.92 × 10^−8^
	SEE	0.0003	0.0003
	SNE	0.3333	0.3227
Langmuir	$1-{\bar{R}}^{2}$	0.2231	0.1693
	$r\chi^{2}$	2.94 × 10^−7^	1.88 × 10^−7^
	SEE	0.0005	0.0004
	SNE	0.8260	0.6216
Freundlich	$1-{\bar{R}}^{2}$	0.0639	0.06
	$r\chi^{2}$	8.42 × 10^−8^	6.68 × 10^−8^
	SEE	0.0003	0.0003
	SNE	0.3407	0.3266
Jovanovic	$1-{\bar{R}}^{2}$	0.1201	0.0825
	$r\chi^{2}$	1.58 × 10^−7^	9.18 × 10^−8^
	SEE	0.0004	0.0003
	SNE	**0.2204**	**0.1405**

**Table 6:** Isotherm error deviation estimation related to the adsorption of atrazine using alternative statistical tools.

Model	Test	MIP Tol:DMSO 9:1	NIP Tol:DMSO 9:1
Linear	*t*-test	0.898998	0.961412
	*F*-test	0.76917	0.899847
Langmuir	*t*-test	0.846388	0.902265
	*F*-test	0.599246	0.743761
Freundlich	*t*-test	0.996748	0.994430
	*F*-test	0.967205	0.982704
Jovanovic	*t*-test	0.999981	0.999997
	*F*-test	0.972053	0.973070

**Table 7:** The parameters of the kinetic adsorption model.

Model	Par.	MIP Tol:DMSO 9:1			
		c_0_ = 2 ppm	c_0_ = 5 ppm	c_0_ = 10 ppm	c_0_ = 20 ppm
B_e_, g_s_/g_pol_		1.85 × 10^−4^	4.48 × 10^−4^	9.80 × 10^−4^	2.88 E× 10^−3^
1st ord.	k_1_	1.36 × 10^2^	1.25 × 10^2^	1.53 × 10^2^	7.50 × 10^1^
2nd ord.	k_2_·B_e_	1.49 × 10^2^	3.87 × 10^2^	5.39 × 10^2^	9.39 × 10^1^
Elovich	α	2.88 × 10^14^	3.65 × 10^15^	1.72 × 10^10^	1.96 × 10^7^
	β	2.34 × 10^5^	1.04 × 10^5^	3.51 × 10^4^	1.00 × 10^4^
		NIP Tol:DMSO 9:1			
B_e_, g_s_/g_pol_		1.96 × 10^−4^	4.24 × 10^−4^	9.49 × 10^−4^	2.88 × 10^−3^
1st ord.	k_1_	8.01 × 10	4.09 × 10^−4^	8.72 × 10^−4^	2.66 × 10^−3^
2nd ord.	k_2_·B_e_	1.15 × 10^1^	2.87 × 10^2^	1.33 × 10^−1^	9.24 × 10^−2^
Elovich	α	1.21 × 10^−2^	3.65 × 10^−5^	5.02 × 10^−5^	3.31 × 10^−4^
	β	3.44 × 10^4^	5.14 × 10	4.38 × 10^3^	1.44 × 10

**Table 8:** Parameter values for determination of heterogeneous electron transfer rate constant, 𝑘d.

Parameter	Value
F(C/mol)	96486
n	1
α	−0.0819
K (anodic)	0.0923
K (cathodic)	1.9101
R(J/K·mol)	8.314
T(K)	303
*E_e_ (V)*	0.2415
Diffusion coefficient of oxidized redox (cm^2^·s^−1^)	5.4 × 10^−3^
Diffusion coefficient of reduced redox (cm^2^·s^−1^)	3.2 × 10^−3^

**Table 9:** Comparison of the performance of various atrazine sensors.

Atrazine Sensor	Limit of Detection (nM)
Mercury film [33]	0.1
BSA/anti-atrazine/GNPs/gold [34]	0.074
Boron-doped diamond [35]	10
Poly(JUG-HATZ) [36]	0.001
Bismuth film [37]	140
MIT/CA [38]	8
Hanging mercury drop [39]	21
MIP/GFE (This work)	4.99

## Data Availability

No new data were created or analyzed in this study. Data sharing is not applicable to this article.

## Nomenclature

**Atrazine Bound per Gram of Polymer**	
C	Capacitance
F_e_	Unbound atrazine per volume of solution
IP	Peak current
*I_pa_*	Anodic current
*I_pc_*	Cathodic current
*K*	Rate of adsorption
K_d_	Equilibrium dissociation constant
M	Molar
*n_F_*	Freundlich isotherm parameter
*N_J_*	Binding site density
*Q*	Template bound
Q	Constant phase element
*Q_max_*	Maximum template bound
R	Resistance
*r^2^*	Coefficient of determination
*R_ct_*,	Charge transfer resistance
R_Ω_	Ohmic resistance
V	Voltage
𝐸_𝑝𝑎_	Anodic peak potential
𝐸_𝑝𝑐_	Cathodic peak potential
𝑘_d_	Heterogeneous electron transfer rate
**Greek Letters**	
α	Transfer coefficient
υ	Scan rate
